# Optimal combination of cow and quinoa milk for manufacturing of functional fermented milk with high levels of antioxidant, essential amino acids and probiotics

**DOI:** 10.1038/s41598-023-47839-6

**Published:** 2023-11-24

**Authors:** Reham Kamal El-Menawy, Doaa Mamdoh Mohamed, Magdy Mohamed Ismail, Amina Mahmoud Hassan

**Affiliations:** 1 Dairy Technology Research Department, Animal Production Research Institute, Agricultural Research Center, Dokki, Giza, Egypt; 2 Dairy Microbiology Research Department, Animal Production Research Institute, Agricultural Research Center, Dokki, Giza, Egypt

**Keywords:** Biotechnology, Microbiology

## Abstract

The aim of this research was to produce Rayeb milk, a bio-fermented milk product that has important benefits for health and nutrition. The Rayeb milk was divided into five different treatments: T1 from cow milk, T2 from quinoa milk, T3 from a mixture of cow and quinoa milk (50%:50%), T4 from a mixture of cow and quinoa milk (75%:25%), and T5 from a mixture of cow and quinoa milk (25%:75%). As a starting culture, ABT-5 culture was used. The results demonstrated that blending quinoa milk with cow milk increased the total solids, fat, total protein, pH, acetaldehyde, and diacetyl values of the resulting Rayeb milk. Additionally, the total phenolic content, antioxidant activity, minerals, and amino acids—particularly important amino acids—in Rayeb milk with quinoa milk were higher. In Rayeb milk prepared from a cow and quinoa milk mixture, *Lactobacillus acidophilus* and *Bifidobacterium bifidum* were highly stimulated. All Rayeb milk samples, particularly those that contained quinoa milk, possessed more bifidobacteria than the recommended count of 10^6^ cfu g^−1^ for use as a probiotic. Based on the sensory evaluation results, it is possible to manufacture a bio-Rayeb milk acceptable to the consumer and has a high nutritional and health values using a mixture of cow milk and quinoa milk (75%:25% or 50%:50%) and ABT-5 culture.

## Introduction

One of the most common dairy products that are naturally fermented in the Middle East is Rayeb milk. It is typically produced as a by-product of manufacturing butter from buffalo milk. The traditional method of making Rayeb milk relies on the spontaneous fermentation of raw milk. The presence and activity of microbes and their enzymes in raw milk led to this fermentation. For around two days, raw milk is left at room temperature before the cream layer is separated and used to make butter and butter oil. Due to the remaining milk's extremely high acid level, it coagulated when heated, producing traditional Rayeb milk. At recent years, large-scale production of safe and standardized Rayeb milk has been carried out at dairy production plants that use ABT culture^1^.

On the other hand, pseudocereals have shown promise and may be used as grains in dietary plans to regulate blood pressure and diabetes^2^. A pseudocereal from the Andes region of South America, primarily Bolivia and Peru, is called quinoa^3^. It is an excellent example with a lot of fiber, making up roughly 2.6–10% of the grain's weight overall, with about 78% of the fiber content being insoluble and 22% being soluble^4,5^. Additionally, vitamins including riboflavin, thiamin, niacin, ascorbic acid, -tocopherol, and -carotene, as well as unsaturated fatty acids like linoleic (52%) and oleic acid (25%), phenolic and flavonoid components, and also, antioxidant, antibacterial properties, and the minerals (potassium, calcium, magnesium, phosphorus, sulphur, iron, and zinc) are all present in quinoa, making it a great source of these nutrients^6,7^. It is one of the most important components is protein which ranges in quinoa from 12 to 16.5% and contains all ten essential amino acids^8^. According to Valencia-Chamorro^9^, quinoa is regarded as a gluten-free grain since it contains little to no prolamin, making it suitable for people with celiac disease or other forms of gluten intolerance.

However, no comprehensive studies about quinoa's use in the production of dairy products could be discovered in the literature. Pineli et al.^10^ produced quinoa milk and shown its benefit for diabetics due to its lower glycemic index. According to Ramrez-Rojas et al.^11^, adding 1.5% of quinoa flour to kefir decreased the fermentation time from 2 to 5 h compared to the control. This is because quinoa flour contains highly fermentable poly saccharides that accelerate the pH's decline. Mabrouk and Effat^12^ supplemented yoghurt with probiotics and a bioactive cereal compound (quinoa flour) to enhance its therapeutic and nutritional function, and they demonstrated that the high nutritional value of quinoa flour improved the yoghurt starter and probiotic counts throughout the refrigeration storage period. So, the aim of this study was to produce bio-Rayeb milk had high nutritional and health values at the same time had desirable sensory attributes. To achieve this purpose, bio-Rayeb milk was made using cow milk, quinoa milk and ABT-5 culture.

## Materials and methods

### Materials

The Animal Production Research Institute in Egypt provided the raw cow milk. From the Egyptian Company for Natural Oils in Cairo, Egypt, quinoa seeds were purchased. Chr. Hansen, lab A/S Copenhagen, Denmark provided the ABT-5 culture, which is formed up of *Streptococcus thermophilus*, *Lactobacillus acidophilus*, and *Bifidobacterium bifidum*.

### Methods

#### Quinoa milk preparation

The quinoa seeds were thoroughly cleaned of contaminants before being soaked in pre-boiled water for 24 h at 25 °C. Water was changed every 8 h. The seeds and water were blended together at a ratio of 1 seed to 6 waters. The quinoa milk that resulted was heated to 90 °C for 10 min, and then cooled to 40 °C.

#### Bio-Rayeb milk preparation

From a blend of cow and quinoa milk, five treatments of Rayeb milk have been produced as follows:

- Rayeb made from only 100% cow's milk is the first treatment (T1)
- Rayeb from 100% quinoa milk is the second treatment (T2)
- Rayeb from 50% cow milk and 50% quinoa milk is the third treatment (T3)
- Rayeb from 75% cow milk and 25% quinoa milk is the fourth treatment (T4)
- Rayeb from 25% cow milk and 75% quinoa milk is the fifth treatment (T5).

Cow milk and quinoa milk were heated to 90 °C for 10 min and then immediately cooled to 40 °C. For samples 3, 4, and 5, cow milk and quinoa milk were mixed. All milk samples were then inoculated with ABT-5 cultures (0.1 g/L of milk), incubated at 40 °C for complete coagulation, and then stored at 4 °C overnight. Once blended for five min and divided into three portions transferred to three jars and kept at 4 °C for 14 days. Fresh Rayeb milk samples were examined, as well as samples after 7, and 14 days of refrigeration. Three replicates of each treatment were conducted.

#### Chemical analysis

For determining the total solids (TS), fat and total protein (TP) contents of samples, AOAC^13^ methods were employed. Using a pH meter (Corning pH/ion analyzer 350, Corning, NY), the pH values were determined. According to Lees and Jago^14^, the acetaldehyde and diacetyl contents of Rayeb samples were determined. The total phenolic content (TPC) of the sample was determined using the Folin-Ciocalteu assay^15^. Amount of TPC was expressed as milligrams of gallic acid equivalents (GAE) per 100 g of sample. Using the stable radical DPPH, the antioxidant activity of Rayeb milk was evaluated in terms of its capacity to donate hydrogen or scavenge free radicals as described by Politeo et al*.*^16^.

#### Determination of the free amino acids

To determine the amino acid content of fresh Rayeb milk, Walsh and Brown's method^17^ was used.

#### Determination of minerals

The elemental makeup of Rayeb samples was determined using Energy Dispersive X-Ray Analysis (EDX), also known as EDS or EDAX. EDX systems are add-ons for scanning electron microscopes (SEM) or transmission electron microscopes (TEM) that allow the imaging capabilities of the microscope to identify a specific specimen. The information produced by EDX analysis consists of spectra with peaks matching to the components of the sample's actual makeup.

#### Microbiological analysis

Using the methods described by Tharmaraj and Shah^18^, *Lactobacillus acidophilus* and *Streptococcus thermophilus* counts were determined in Rayeb milk samples. Enumeration of *Lactobacillus acidophilus* was done using MRS-sorbitol agar medium and the plates were incubated anaerobically at 37 °C for 48 h whereas the counting of *Streptococcus thermophiles* was determined using M17-lactose agar and the plates were incubated aerobically at 37 °C for 24 h. Bifidobacteria were counted in accordance with Dinakar and Mistry^19^ by using modified MRS agar supplement with 0.05% l-cystein and 0.3% Lithium chloride. The plates were incubated at 37 °C for 48 h under anaerobic condition.

#### Evaluation of sensory attributes

A panel of judges who were familiar with the product evaluated the sensory qualities of Rayeb milk treatments using the hedonic scale, where 1–10 represents strongly dislike to strongly liking^20^.

#### Statistical analysis

Two-way ANOVA was used to statistically analyze the data by SPSS statistics 22.0 in order to determine whether there were any notable variations between the means of the samples and the period of storage. The Tukey test was used to compare the means of the results at a significance level of 5% (p < 0.05). The mean and standard deviation of three replicates were used to express all data.

### Ethical approval

All the steps of experimentation on quinoa plant, including the collection of plant material, are in compliance with relevant Institutional, National, and International guidelines. The different studies were conducted in accordance with local legislation and with permissions from our institutes and complied with the IUCN Policy Statement.

## Results and discussion

### The physical and chemical properties of the cow milk and quinoa milk used for producing Rayeb

The chemical properties of cow's milk generally were within the normal ranges for its chemical composition (acidity 0.17%, pH 6.61, total solids 13.03, fat 4.3, and total protein 3.38%).

Acidity was 0.23%, pH was 5.92, total solids were 16.20, fat was 5.2, and total protein was 4.93% in quinoa milk. Quinoa milk had a low pH and higher levels of acidity than cow milk. These results are similar to those found by El-Deeb et al*.*^21^ who stated that the acidity and pH values of quinoa seeds water extract were 0.26% and 3.51 respectively. In our study, for keeping all nutritional value of quinoa, quinoa milk was prepared without filtration, just grinding of soaked seeds with water (1:6 *w/v*) which led to an increase in the levels of TS, fat and protein in the resulted milk compared to what was reported in other literature. The total solids, fat and protein concentrations of quinoa milk were 16.20, 5.90 and 4.93% respectively. Kaur and Tanwar^22^ prepared quinoa milk by grinding soaked seeds with water (1:6 *w/v*) and filtration of milk. Milk had a 0.81 and 1.2% fat and protein contents, respectively. Also, the aqueous extract of quinoa made by Bianchi et al*.*^23^ had lower components level (TS 5.10%, fat 0.11% and protein 0.80%) than that obtained in this study. The differences could be explained by the quinoa extract's high dilution (1:18 w/v).

### Physicochemical properties of Raybe milk

Data of Table 1 show the chemical composition of Rayeb milk during storage period. Blending quinoa milk with cow milk resulted in reducing of the pH values of Rayeb milk. Additionally, Rayeb treatments with the highest levels of quinoa milk showed the strongest evidence of the pH values lowering during the period of storage. According to Codină et al.^24^, adding quinoa flour to yoghurt causes the acidity to increase and pH values to decrease. In the same direction, Mabrouk and Effat^12^ reported that the addition of quinoa flours up to 1% caused a decrease in pH values and an increase in yoghurt acidity. According to Table 1, sample T2 (quinoa milk) had the highest total solid content, which was followed by sample T5 (25% cow milk + 75% quinoa milk), whereas treatment T1 (cow milk) had the lowest value. The high fat content of quinoa seeds^7^ led to significant increasing (P < 0.05) in fat concentrations of Rayeb milk, particularly T3 (50% cow milk + 50% quinoa milk) and T5 samples. Additionally, the high protein content of quinoa milk increased this component in Rayeb milk, which resulted in measurements of 4.99, 6.27, 5.84, 5.17, and 6.09%, respectively, in T1, T2, T3, T4, and T5, at the ending of the storage period.

**Table 1:** Physicochemical properties of Rayeb milk made from mixture of cow and quinoa milk.

Properties	Treatments	Storage period (day)			
		1	7	14	Means ± SD
pH	T1	4.51 ± 0.01	4.33 ± 0.01	4.25 ± 0.01	4.36 ± 0.13^A^
	T2	4.40 ± 0.01	4.20 ± 0.02	4.09 ± 0.04	4.23 ± 0.14^C^
	T3	4.47 ± 0.03	4.28 ± 0.10	4.18 ± 0.01	4.31 ± 0.15^AB^
	T4	4.48 ± 0.02	4.30 ± 0.03	4.22 ± 0.02	4.33 ± 0.17^A^
	T5	4.42 ± 0.02	4.23 ± 0.09	4.13 ± 0.02	4.26 ± 0.14^BC^
	Means ± SD	4.46 ± 0.06^a^	4.25 ± 0.08^b^	4.18 ± 0.08^c^	
TS%	T1	14.58 ± 0.01	14.60 ± 0.05	14.61 ± 0.01	14.59 ± 0.06^E^
	T2	17.24 ± 0.04	17.22 ± 0.02	17.25 ± 0.05	17.24 ± 0.04
	T3	16.07 ± 0.02	16.10 ± 0.05	16.15 ± 0.56	^A^16.00 ± 0.32
	T4	15. 33 ± 0.03	15.39 ± 0.10	15.40 ± 0.04	^C^15.37 ± 0.06
	T5	16.67 ± 0.07	16.65 ± 0.10	16.60 ± 0.10	^D^16.64 ± 0.08^B^
	Means ± SD	15.98 ± 0.09^a^	16.06 ± 0.92^a^	15.83 ± 0.14^b^	
Fat%	T1	4.50 ± 0.05	4.60 ± 0.05	4.50 ± 0.10	4.53 ± 0.07^E^
	T2	5.30 ± 0.10	5.30 ± 0.03	5.30 ± 0.10	5.30 ± 0.07^A^
	T3	4.80 ± 0.02	4.80 ± 0.10	4.70 ± 0.05	4.77 ± 0.08^C^
	T4	4.70 ± 0.02	4.70 ± 0.06	4.70 ± 0.07	4.70 ± 0.05^D^
	T5	5.00 ± 0.05	5.10 ± 0.08	5.00 ± 0.10	5.03 ± 0.08^B^
	Means ± SD	4.86 ± 0.29^b^	4.91 ± 0.26^a^	4.82 ± 0.31^b^	
TP%	T1	4.89 ± 0.01	4.88 ± 0.20	4.99 ± 0.30	4.92 ± 0.19^E^
	T2	6.22 ± 0.22	6.26 ± 0.10	6.27 ± 0.02	6.25 ± 0.12^A^
	T3	5.80 ± 0.20	5.89 ± 0.01	5.84 ± 0.30	5.84 ± 0.19^C^
	T4	5.28 ± 0.10	5.20 ± 0.05	5.17 ± 0.03	5.22 ± 0.08^D^
	T5	6.05 ± 0.05	6.04 ± 0.19	6.09 ± 0.10	6.04 ± 0.14^B^
	Means ± SD	5.65 ± 0.53^a^	5.69 ± 0.54^a^	5.61 ± 0.56^a^	

^abcd^Letters indication to significant differences between the samples of Rayeb ± SD; ^ABCD^ letters indication to significant differences between storage period of Rayeb ± SD; T1: Rayeb from 100% cow milk; T2: Rayeb from 100% quinoa milk; T3: Rayeb from 50% cow milk + 50% quinoa milk; T4: Rayeb from 75% cow milk + 25% quinoa milk; T5: Rayeb from 25% cow milk + 75% quinoa milk.

Table 2 shows the effects of quinoa milk addition on the acetaldehyde and diacetyl content of Rayeb milk. The highest acetaldehyde content was found in quinoa milk Rayeb (T2) (44.58 ppm). As a result, adding quinoa milk to cow milk considerably (P < 0.05) increased the acetaldehyde content in various Rayeb samples. The positive effect of quinoa milk on acetaldehyde content of Rayeb may be credited to the stimulation impact of quinoa on the starter culture during fermentation period. As is well-known, the activity of microorganisms in starting cultures has an impact on the production of volatile components. Another explanation for an increase in quinoa Rayeb's acetaldehyde content is that the addition of quinoa increased the protein level, which affects the content of flavoring compounds. According to Saint-Eve et al.^25^, the protein content of yoghurt has an impact on the production of carbonyl compounds. Many flavoring substances interact with proteins through reversible and irreversible binding.

**Table 2:** Acetaldehyde, diacetyl, total phenolic content (TPC) and antioxidant contents of Rayeb milk made from mixture of cow and quinoa milk.

Properties	Treatments	Storage period (day)			
		1	7	14	Means ± SD
Acetaldehyde (ppm)	T1	39.83 ± 0.03	19.35 ± 0.1	8.03 ± 0.03	22.4 ± 0.05^E^
	T2	44.58 ± 0.10	26.49 ± 0.1	15.94 ± 0.04	29.00 ± 0.08^A^
	T3	41.90 ± 0.10	22.81 ± 0.01	12.15 ± 0.05	25.62 ± 0.05^C^
	T4	40.51 ± 0.20	19.96 ± 0.11	9.29 ± 0.10	23.25 ± 0.13^D^
	T5	42.63 ± 0.03	23.37 ± 0.20	15.65 ± 0.1	27.22 ± 0.11^B^
	Means ± SD	41.89 ± 1.73^a^	22.39 ± 2.66^b^	12.21 ± 3.33^c^	
Diacetyl (ppm)	T1	9.53 ± 0.10	12.34 ± 0.20	7.84 ± 0.04	9.90 ± 1.97^E^
	T2	16.58 ± 0.05	21.89 ± 0.10	15.98 ± 0.02	18.15 ± 2.8^A^
	T3	13.64 ± 0.04	17.81 ± 0.01	13.56 ± 0.04	15.00 ± 2.10^C^
	T4	11.14 ± 0.14	14.34 ± 0.25	10.48 ± 0.30	11.99 ± 1.81^D^
	T5	15.78 ± 0.01	21.03 ± 0.03	15.16 ± 0.10	17.32 ± 2.79^B^
	Means ± SD	13.33 ± 2.77^b^	17.48 ± 3.83^a^	12.60 ± 3.14^c^	
TPC (mg GAE/100 g of sample)	T1	8.69 ± 0.10	11.12 ± 0.10	5.09 ± 0.09	8.32 ± 2.63^E^
	T2	27.31 ± 0.30	33.44 ± 0.10	26.52 ± 0.10	29.09 ± 3.28^A^
	T3	17.25 ± 0.25	21.29 ± 0.01	14.32 ± 0.02	17.62 ± 3.03^C^
	T4	11.91 ± 0.01	14.18 ± 0.10	7.21 ± 0.20	11.10 ± 3.08^D^
	T5	21.34 ± 0.20	26.40 ± 0.10	20.43 ± 0.10	22.73 ± 2.78^B^
	Means ± SD	17.31 ± 6.84^b^	21.28 ± 8.38^a^	14.71 ± 8.29^c^	
Antioxidant (DPPH inhibition %)	T1	49.53 ± 0.03	53.0 ± 0.50	44.04 ± 0.04	48.85 ± 3.92^E^
	T2	60.53 ± 0.03	72.7 ± 0.72	56.61 ± 0.01	63.21 ± 7.17^A^
	T3	54.48 ± 0.10	58.6 ± 0.10	47.65 ± 0.02	53.56 ± 4.80^C^
	T4	50.83 ± 0.20	57.09 ± 1.0	44.86 ± 0.10	50.92 ± 5.32^D^
	T5	58.63 ± 0.03	66.30 ± 0.3	53.12 ± 0.10	59.35 ± 5.73^B^
	Means ± SD	54.80 ± 4.42^b^	61.49 ± 7.25^a^	29.24 ± 5.03^c^	

^abcd^Letters indication to significant differences between the samples of Rayeb ± SD; ^ABCD^ letters indication to significant differences between storage period of Rayeb ± SD; T1: Rayeb from 100% cow milk; T2: Rayeb from 100% quinoa milk; T3: Rayeb from 50% cow milk + 50% quinoa milk; T4: Rayeb from 75% cow milk + 25% quinoa milk; T5: Rayeb from 25% cow milk + 75% quinoa milk.

The results presented in Table 2 for diacetyl values showed that its production followed the same trend as acetaldehyde production. Compared to the control, the quinoa milk in Rayeb had higher diacetyl levels. These results are consistent with those of Mabrouk and Effat^12^, who stated that adding quinoa flour to bio-yogurt increased its diacetyl amount. Generally, in all Rayeb treatments the acetaldehyde values gradually lowered during storage period. On the contrary, diacetyl content increased gradually up to the seventh day of storage, then reduced as storage period progressed. Because acetaldehyde is rapidly converted to acetate at lower pH values, its level decreases with storage^26^. Lactic acid starter cultures have the ability to convert acetaldehyde to diacetyl and ethanol, as demonstrated by Blassy and Abdeldaiem^27^.

### Antioxidant activity and total phenolic content (TPC) in Rayeb milk

As cleared in Table 2, the TPC of various Rayeb milk treatments correlated with their antioxidant activity. Similar trend was obtained by Kaur and Tanwar^22^. Rayeb milk made from quinoa milk (T2) exhibited the highest TPC and inhibition of DPPH oxidation activity. The least levels belonged to control sample (T1). The antioxidant activity and TPC of Rayeb milk were both considerably (P < 0.05) increased by adding quinoa to cow milk. Lorusso et al.^28^ found a comparable influence for the barley, oat, and quinoa substrates used for lactic acid fermentation. According to several research^22,29^, quinoa seeds are a great source of antioxidants and TPC content has a direct impact on antioxidant activity. According to research by Karoviová et al.^30^, fermentation of quinoa beverages with commercially available probiotic culture containing *Bifidobacterium *sp., *Lactobacillus acidophilus*, and *Streptococcus thermophilus* significantly increased the total phenolic content and antioxidation activity in the final products.

The TPC and antioxidant activity increased in all Rayeb treatments up to the seventh day of storage, then gradually decreased as storage time extended. According to Amirdivani and Baba^31^, increased degradation of phenolic compounds with antioxidant activities and/or increased milk protein polyphenol interaction may be responsible for a decrease in antioxidant activities during the refrigeration of yoghurt.

### Mineral content of Rayeb milk

**Figure 1:**
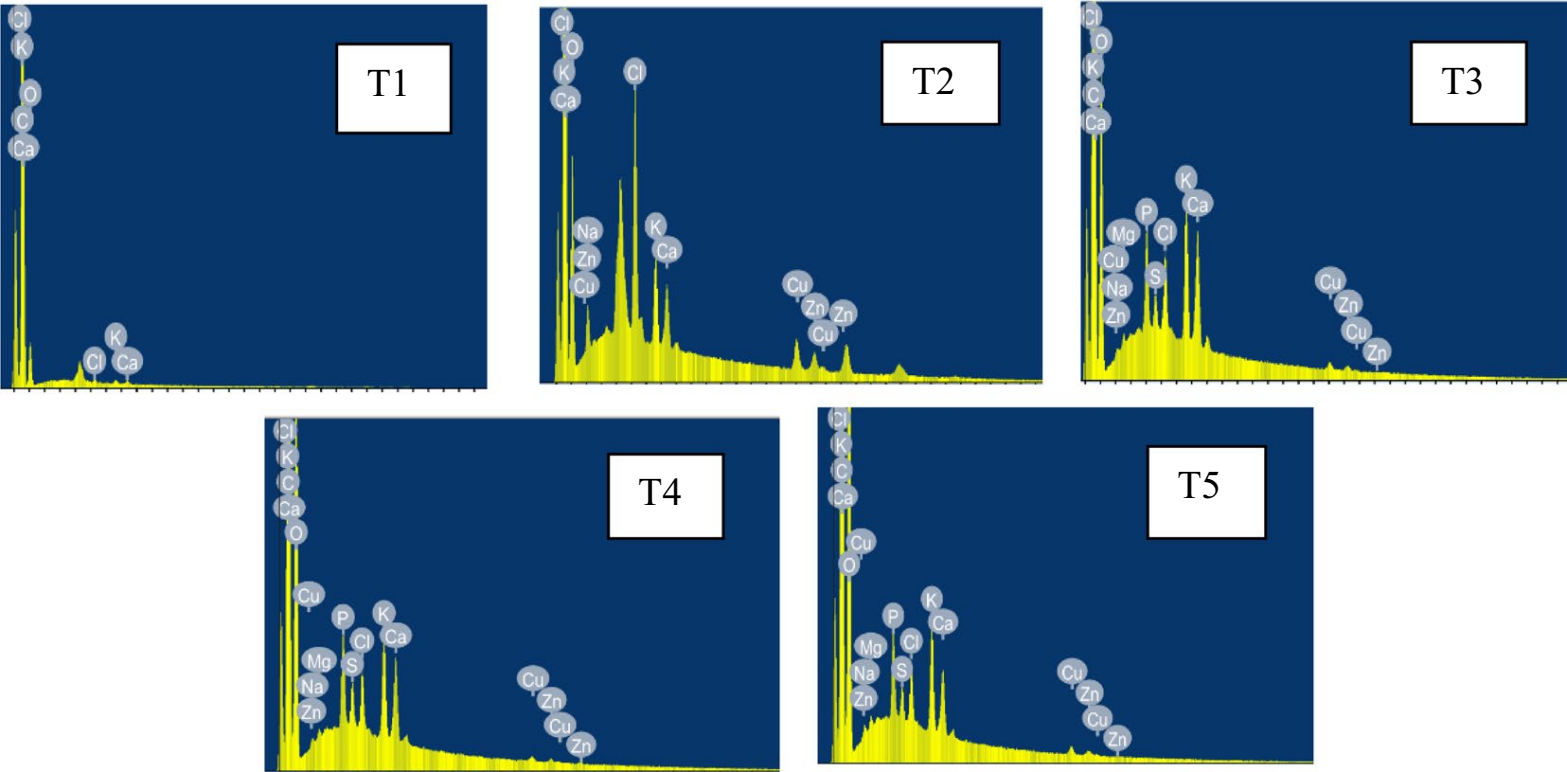
The mineral Content of Rayeb milk at the beginning of storage period.

Figure 1 displayed the mineral composition for various fresh Rayeb milk treatments. The amounts of Na, S, Cl, Ca, and Zn in Rayeb milk increased after cow milk was fortified with quinoa milk. In contrast, quinoa milk Rayeb possessed the lowest contents of P and K. No difference among Rayeb samples regarding their Mg and Cu contents. El-Deeb et al.^21^ observed that as the amount of quinoa seeds water extract (QSWE) was increased, the minerals content of fermented quinoa beverages derived from buffalo skim milk decreased. According to Abd-Rabou et al.^32^, Kishk (an artisanal fermented popular food in the Middle East) prepared from quinoa and wheat grains with camel milk and probiotic culture displayed the highest content of iron as compared with control made from wheat grains with cow milk and normal starter culture. This is mostly because the formula contains camel milk and quinoa grains. Similar to this pattern, Ismail and Rayan^33^ found that the mineral content of Kishk samples increased as the amount of quinoa seeds in the Kishk formulation increased. K, Mg, and Ca levels were higher in the Kishk sample (100% quinoa seeds) than in the control (100% wheat burghul).

### Fresh Rayeb milk's free amino acid content (FAA)

The results presented in Table 3 show the impact of combining quinoa milk with cow milk on the FAA content of fresh Rayeb milk. The largest levels of essential and non-essential amino acids were found in Rayeb produced from quinoa milk (T2), which had 4.708 and 3.677 g/100 ml, respectively. As a result, the produced Rayeb milk's amino acid composition was improved by fortifying cow milk with quinoa milk. For samples T3, T4, and T5, adding quinoa milk to Rayeb increased the amount of essential amino acids by 3.15, 1.31, and 8.99%, respectively. As is well known, the ratio of necessary amino acids that cannot be synthesized by living beings determines the nutritional quality of protein in the diet^34^. Subsequently, increasing of essential amino acids levels of Rayeb by adding quinoa milk raised its nutritional value. The protein content of quinoa seeds is major and they also contain essential amino acids, sulphur amino acids, and amino acids^34^. Approximately 180% of the histidine, 274% of the isoleucine, 338% of the lysine, 212% of the methionine, and 323% of the valine needed in protein sources for adult nutrition can be found in quinoa protein, per data^35^.

**Table 3:** Free amino acids content (g/100 mL) of fresh Rayeb milk.

Amino acids	Treatments				
	T1	T2	T3	T4	T5
Essential amino acids					
Cysteine	0.040	0.037	0.039	0.040	0.040
Histidine	0.377	0.425	0.401	0.382	0.424
Isoleucine	0.458	0.449	0.456	0.448	0.451
Leucine	0.717	0.733	0.719	0.717	0.736
Lysine	0.834	0.863	0.842	0.838	0.870
Methionine	0.181	0.192	0.185	0.181	0.194
Phenylalanine	0.641	0.667	0.647	0.645	0.668
Threonine	0.379	0.492	0.430	0.408	0.508
Tyrosine	0.437	0.485	0.454	0.449	0.477
Valine	0.284	0.365	0.312	0.297	0.371
Total	4.348	4.708	4.485	4.405	4.739
Nonessential amino acids					
Alanine	0.367	0.391	0.379	0.371	0.390
Arginine	0.316	0.477	0.391	0.339	0.504
Aspartic acid	0.488	0.605	0.545	0.505	0.599
Glutamic acid	0.823	0.948	0.848	0.837	0.928
Glycine	0.079	0.090	0.083	0.080	0.088
Proline	0.746	0.751	0.742	0.750	0.760
Serine	0.415	0.415	0.419	0.417	0.424
Total	3.234	3.677	3.407	3.299	3.693

T1: Rayeb from 100% cow milk; T2: Rayeb from 100% quinoa milk; T3: Rayeb from 50% cow milk + 50% quinoa milk; T4: Rayeb from 75% cow milk + 25% quinoa milk; T5: Rayeb from 25% cow milk + 75% quinoa milk.

Lysine had the highest concentration of all the essential amino acids, followed by leucine, while cysteine had the lowest quantity. Of the non-essential amino acids, glutamic acid had the highest value while glycine had the lowest. In all Rayeb treatments, the concentration of essential amino acids was higher than the concentration of nonessential amino acids.

### Starter bacteria numbers of Rayeb milk

Table 4 shows counts of *S. thermophilus, L. acidophilus*, and *Bifidobacterium bifidum* during the period of storage of Rayeb milk. The results showed an increase in numbers of the mentioned microorganisms during the first seven days, then their populations decreased. This could be due to the bacterial activity, which causes an increase in acidity in the medium, making it unsuitable for bacterial growth or acting as a bactericidal agent.

**Table 4:** Starter bacteria counts of Rayeb milk made from mixture of cow and quinoa milk.

Properties	Treatments	Storage period (day)			
		1	7	14	Means ± SD
*Streptococcus thermophiles* (10^5^ CFU/mL)	T1	45 ± 1.0	51 ± 1.0	42 ± 2.0	46.0 ± 4.15^D^
	T2	53 ± 3.0	68 ± 4.0	60 ± 5.0	60.33 ± 7.39^AB^
	T3	50 ± 5.0	62 ± 2.0	57 ± 2.0	56.33 ± 5.96^BC^
	T4	44 ± 1.0	58 ± 2.0	55 ± 5.0	52.33 ± 6.95^C^
	T5	56 ± 6.0	69 ± 1.0	62 ± 2.0	60.44 ± 17.23^AB^
	Means ± SD	50.26 ± 7.56^c^	61.60 ± 7.15^a^	55.2 ± 7.85^b^	
*Lactobacillus acidophilus* (10^5^ CFU/mL)	T1	29 ± 1.0	41 ± 10.0	28 ± 2.0	32.67 ± 8.09^E^
	T2	38 ± 6.0	51 ± 10.0	42 ± 2.0	43.89 ± 8.10^AB^
	T3	35 ± 5.0	47 ± 3.0	36 ± 3.0	39.22 ± 6.49^CD^
	T4	33 ± 3.0	45 ± 1.0	33 ± 1.0	37.0 ± 6.22^DE^
	T5	40 ± 2.0	51 ± 10.0	44 ± 6.0	45.0 ± 7.63^A^
	Means ± SD	35.13 ± 5.25^b^	46.93 ± 7.73^a^	36.6 ± 6.66^b^	
*Bifidobacterium bifidum* (10^5^ CFU/mL)	T1	35 ± 2.0	46 ± 6.0	34 ± 4.0	38.33 ± 6.87^D^
	T2	47 ± 3.0	59 ± 1.0	51 ± 4.0	52.33 ± 5.87^A^
	T3	40 ± 2.0	55 ± 5.0	44 ± 4.0	46.33 ± 7.52^B^
	T4	38 ± 6.0	49 ± 1.0	39 ± 3.0	42.33 ± 6.1^C^
	T5	50 ± 10.0	60 ± 5.0	52 ± 2.0	54.0 ± 7.29^A^
	Means ± SD	42.13 ± 7.39^b^	53.80 ± 6.69^a^	44.06 ± 7.71^b^	

^abcd^Letters indication to significant differences between the samples of Rayeb ± SD; ^ABCD^ letters indication to significant differences between storage period of Rayeb ± SD; T1: Rayeb from 100% cow milk; T2: Rayeb from 100% quinoa milk; T3: Rayeb from 50% cow milk + 50% quinoa milk; T4: Rayeb from 75% cow milk + 25% quinoa milk; T5: Rayeb from 25% cow milk + 75% quinoa milk.

Results shown in Table 4 showed that *S. thermophiles* were more prevalent in Rayeb milk prepared from quinoa or a combination of cow milk and quinoa milk than in the control sample, which had the lowest count. Because quinoa Rayeb milk is rich in nutrients like minerals and amino acids, which promote the growth of starting culture, this is exactly what was expected. As a result, the functional properties of the resultant Rayeb are enhanced, and quinoa may be used as a prebiotic. Because it contains a lot of minerals and amino acids, quinoa flour promotes the formation of yoghurt starter cultures and probiotic bacteria^36^. According to Karoviová et al.^30^, quinoa is an appropriate substrate for lactic acid fermentation.

Table 4 shows that the addition of quinoa milk to cow milk had a significant impact on the activity and count of *Lactobacillus acidophilus* in Rayeb milk. Samples T2 (quinoa milk) and T5 (25% cow milk + 75% quinoa milk) had the highest counts of *L. acidophilus* through the storage time. This indicates that *L. acidophilus* might grow and leave a good impression on quinoa Rayeb. According to Casarotti et al.^37^, adding quinoa flour to milk increased the population of *L. acidophilus La-5* and *B. animalis ssp. lactis BB-12* at 28 days of fermented milk storage compared to the control treatment. The chemicals included in quinoa flour may be responsible for the probiotic strain's improved development in fermented milk that contained 3% quinoa flour. High levels of carbohydrate, fibre, linoleic, -linolenic, and oleic acids, as well as folate, potassium, phosphorus, magnesium, calcium, iron, and zinc, are characteristics of quinoa flour. Due to the high food requirements of probiotic microorganisms, these substances aid in their growth during the fermentation of milk storage.

Bifidobacteria numbers had the same trend of *S. thermophilus* and *L. acidophilus*. The greatest numbers of bifidobacteria, particularly in samples T2 and T5, were seen in Rayeb milk that contained quinoa. Not only that, but the viability loss of *Bifidobacterium bifidum* during the period of the last seven days of storage were lower in the quinoa samples than they were in the control, resulting in at 26.08, 13.56, 20.00, 20.41, and 13.33% for samples T1, T2, T3, T4, and T5, respectively. The activating effect of quinoa components on *Bifidobacterium bifidum* is reflected in these studies. According to Mabrouk and Effat^12^, the high nutrient content and components of quinoa flour could increase the survival and high probiotic counts in yoghurt.

To have the positive probiotic impact, the probiotic bacteria count must be over 10^6^ cfu g^−1^^38^. However, probiotic bacteria populations decreased during storage but they remained over 10^6^ cfu g^−1^ in various Rayeb milk treatments. Rayeb milk particularly that contained quinoa milk had a beneficial probiotic impact. According to Casarotti et al.^37^ quinoa flour may have provided probiotics some protection against gastric and enteric juices during their simulated passage through the GI tract. This is because it acts as a defense against these acids. As a result, it is possible that flour components (protein, lipids, and fiber) improved probiotic tolerance to simulated GI conditions. Additionally, Abd-Rabou et al.^32^ demonstrated that adding wheat or quinoa to cow and camel milk to make kishk helps in preventing undesirable microbes and preserves the probiotic bacterial count level necessary for exerting health advantages.

### Sensory evaluation of Rayeb milk

Table 5 displays the sensory evaluations of Rayeb milk produced from cow milk, quinoa milk and cow and quinoa milk admixtures. Except for sample T2 (quinoa milk), which recorded the lowest score of color and appearance during the storage period, no differences in color and appearance values between the different Rayeb treatments were found to be statistically significant (P ≤ 0.05). The panelists gave lower color and appearance scores for quinoa Rayeb because of its dark color. Due to the dark color of quinoa, kishk treatments that contained it had a lower color score, but they were still acceptable^32^.

**Table 5:** Sensory evaluation of Rayeb milk made from mixture of cow and quinoa milk.

Properties	Treatments	Storage period (day)			
		1	7	14	Means ± SD
Color and appearance (15)	T1	14 ± 1.0	14 ± 1.0	13 ± 1.0	13.67 ± 1.0^A^
	T2	12 ± 1.0	11 ± 1.0	9 ± 2.0	10.67 ± 1.73^B^
	T3	14 ± 2.0	14 ± 1.0	13 ± 1.0	13.67 ± 1.32^A^
	T4	14 ± 1.0	14 ± 1.0	13 ± 2.0	13.67 ± 1.32^A^
	T5	13 ± 1.0	12 ± 1.0	10 ± 2.0	11.67 ± 1.80^B^
	Means ± SD	13.40 ± 1.29^a^	13.0 ± 1.56^a^	11.60 ± 2.29^b^	
Texture (35)	T1	33 ± 3.0	33 ± 2.0	31 ± 1.0	32.33 ± 2.12^A^
	T2	30 ± 1.0	28 ± 1.0	25 ± 1.0	27.66 ± 2.34^C^
	T3	34 ± 2.0	34 ± 2.0	32 ± 2.0	33.3 ± 2.0^A^
	T4	34 ± 1.0	34 ± 2.0	33 ± 3.0	33.67 ± 1.93^A^
	T5	32 ± 2.0	30 ± 1.0	27 ± 2.0	29.67 ± 2.65^B^
	Means ± SD	32.60 ± 2.26^a^	31.80 ± 2.86^a^	29.60 ± 3.58^b^	
Flavor (50)	T1	48 ± 1.0	47 ± 2.0	45 ± 1.0	46.67 ± 1.80^A^
	T2	42 ± 2.0	39 ± 1.0	35 ± 2.0	38.67 ± 3.39^C^
	T3	48 ± 4.0	47 ± 1.0	45 ± 1.0	46.67 ± 2.50^A^
	T4	48 ± 2.0	46 ± 2.0	45 ± 1.0	46.33 ± 2.0^A^
	T5	44 ± 2.0	41 ± 1.0	38 ± 2.0	41.0 ± 3.0^C^
	Means ± SD	46.0 ± 3.31^a^	44.0 ± 3.68^b^	41.60 ± 459^c^	
Overall acceptability (100)	T1	95 ± 1.0	94 ± 1.0	88 ± 2.0	90.11 ± 1.80^A^
	T2	83 ± 3.0	78 ± 1.0	70 ± 2.0	77.0 ± 5.97^C^
	T3	96 ± 1.0	94 ± 2.0	88 ± 2.0	92.67 ± 3.90^A^
	T4	95 ± 1.0	94 ± 2.0	87 ± 1.0	92.22 ± 4.12^A^
	T5	90 ± 3.0	86 ± 1.0	79 ± 1.0	85.0 ± 5.09^B^
	Means ± SD	91.80 ± 5.33^a^	89.33 ± 6.85^b^	87.40 ± 8.0^c^	

^abcd^Letters indication to significant differences between the samples of Rayeb ± SD; ^ABCD^ letters indication to significant differences between storage period of Rayeb ± SD; T1: Rayeb from 100% cow milk; T2: Rayeb from 100% quinoa milk; T3: Rayeb from 50% cow milk + 50% quinoa milk; T4: Rayeb from 75% cow milk + 25% quinoa milk; T5: Rayeb from 25% cow milk + 75% quinoa milk.

The texture evaluations showed that Rayeb's texture was improved by supplementing it with quinoa milk by up to 50% (T3 and T4). Rayeb made from 100% quinoa milk registered the lowest texture value. According to research by Tang et al.^39^, quinoa protein has a higher water holding capacity (WHC) than proteins from oat, soybean, and wheat, hence it is expected that quinoa will improve its textural properties in a variety of food applications.

According to Table 5 data, Rayeb produced from cow milk (Control, T1), 50% cow milk plus 50% quinoa milk (T3), or 75% cow milk plus 25% quinoa milk (T4), received the highest scores for flavor. Although fermentation enhances the sensory characteristics of cereal beverages, the majority of panelists did not like the flavor of Rayeb, which contained a lot of quinoa (T2 and T5). According to Curti et al.^40^, the aroma and flavor of yoghurt were found to be more unpleasant when quinoa flour was added at increasing amounts.

Because of this, the treatments T1, T3, and T4 obtained high scores for color, appearance, texture, and flavor. They also received the highest scores for overall acceptability both at the start of the evaluation and during the storage period. According to Karoviová et al.^30^, fermented quinoa beverages have a creamy, light color and a sour odor and taste. The raspberry syrup was added as a supplement to beverages since they had a poor level of sensory acceptance. Ismail and Rayan^33^ also noted that the total acceptability of kishk decreased when quinoa seeds were added in amounts more than 50%.

The organoleptic properties scores decreased over the period of storage in all Rayeb milk samples. These results are consistent with those made by Ranadheera et al.^41^, who reported that the sensory acceptability of fermented goat's milk that had been stored for three weeks was lower than that of the comparable fresh products.

## Conclusion

Utilization of quinoa aqueous extracts (quinoa milk) as a health and nutritonaly material in production of bio-Rayeb milk was studied in this investigation. The results cleared that it is possible to manfucture health and nutritonaly bio-Rayeb milk based on cow milk and quinoa milk mixtures (75% + 25% or 50% + 50% respectively) and ABT-5 culture. Rayeb prepared from those mixture possessed high levels of total soilds, fat, protein, acetaldehyde, diacetyl, total phenolic content, antioxidant activity, minerals and essential amino acids. Rayeb contained 25 or 50% quinoa milk had a higher probiotic survival rate and sensory evalutions scores.

## Data Availability

All data generated or analyzed during this study are included in this published article.
